# Psychometric properties of the Adult ADHD Self-Report Scale (ASRS-5) Portuguese version

**DOI:** 10.1192/j.eurpsy.2026.11507

**Published:** 2026-07-31

**Authors:** A. I. Araújo, A. Macedo, M. J. Brito, C. Cabaços, A. T. Pereira

**Affiliations:** Institute of Psychological Medicine, Faculty of Medicine University of Coimbra, Coimbra, Portugal

## Abstract

**Introduction:**

Attention-deficit/hyperactivity disorder (ADHD) is a neurodevelopmental disorder that starts from childhood and lasts through adulthood. Despite its high prevalence and impact, adults ADHD remains undiagnosed and untreated, not only but mainly in women.

The short version of the Adult **ADHD Self-Report Scale (ASRS)** composed of 6 items has been shown to outperform the original 18-item scale. The updated version for *DSM-5* criteria (ASRS-5) has high psychometric and operating characteristics, being recommended for adult cases detection in the general population.

**Objectives:**

To analyse the psychometric properties (construct validity and internal consistency) of the Portuguese version of the ASRS-5.

**Methods:**

792 adults from the general population (71.5% females; 28.5% males), with a mean age of 30.91 (±15.05; range:18-74) years, completed an online survey including the ASRS-5.

**Results:**

The Confirmatory Factor Analysis of the unidimensional model resulted in “good” / “very good” fit indices (χ2/gL=2.772; CFI=.9888; TLI=.9790; GFI=.9115; RMSEA=.0467; p<0.001). Factor loadings ranged from 0.565 to 0.741.

Internal consistency resulted in Raykovs Omega 0.861 and Cronbach’s *α*=0.799.

Total scores were significantly higher in women (7.443±4.426) than in men (6.491±4.334; T Student=-2.750, p=0.006).

**Conclusions:**

ASRS-5 Portuguese version present adequate construct validity and reliability, with results comparable to those found in the original English and other versions. Our results are also in line with growing evidence that this condition may affect women more than men. This psychometric study represents a first contribution to both clinical and research on ADHD in Portugal. In a near future study, we intend to analyse its sensitivity and specificity, to identify the cut-off point with the best accuracy for the Portuguese population. Since the group of Portuguese speakers is larger and more culturally diverse than the Portuguese population, it will also be important to explore the ASRS-5 validity, reliability and screening accuracy with samples from other Portuguese-speaking countries.

**Disclosure of Interest:**

None Declared

